# Predictors of Impaired Postpartum Renal Function in Women after Preeclampsia: Results of a Prospective Single Center Study

**DOI:** 10.1155/2016/7861919

**Published:** 2016-07-31

**Authors:** T. Kaleta, A. Stock, D. Panayotopoulos, O. Vonend, D. Niederacher, M. Neumann, T. Fehm, W. Kaisers, M. Fleisch

**Affiliations:** ^1^Department of Obstetrics and Gynaecology, Heinrich-Heine-University Medical Centre, Moorenstrasse 5, 40225 Duesseldorf, Germany; ^2^Department of Obstetrics and Gynecology, Dormagen Hospital, Dr.-Geldmacher-Straße 20, 41540 Dormagen, Germany; ^3^Department of Nephrology, DKD Helios Clinic Wiesbaden GbR, Aukammallee 33, 65191 Wiesbaden, Germany; ^4^Department of Mathematics, Faculty of Mathematics and Natural Sciences, Heinrich-Heine-University, Universitätsstrasse 1, 40225 Düsseldorf, Germany; ^5^Department of Obstetrics and Gynaecology, Helios University Medical Center Wuppertal, Heusnerstraße 40, 42283 Wuppertal, Germany

## Abstract

*Objective*. The purpose of this prospective study was to investigate the predictive value of single prepartum findings combined with serum biomarkers sFlt-1 (soluble fms-like tyrosine kinase-1) and PlGF (placental growth factor) indicating severity of preeclampsia (PE) for occurrence and extent of impaired postpartum kidney function.* Study Design*. In this prospective, single center study 44 PE patients were compared to 39 healthy controls (similar in age and gestational age with singleton pregnancy) evaluated at time of delivery and at 6 months and 12 months postpartum. *p* values below 0.05 are considered statistically significant.* Results*. The majority of the PE patients had persistence of proteinuria (>120 mg/L after delivery) 6 months (*p* = 0.02) and 12 months postpartum (*p* < 0.0001) compared to controls. Also reduced GFR (glomerular filtration rate) persisted up to 6 months postpartum in PE patients compared to controls (*p* < 0.001). Prepartum sFlt-1 levels indeed correlated with impaired renal function parameters.* Conclusion*. A significant proportion of our PE patients had lower GFR levels and persistent proteinuria up to 12 months postpartum. Prepartum sFlt-1 is a trend-setting marker for impaired renal function postpartum, but it is not sufficient enough to predict renal impairment after PE. An evaluation of 24-month follow-up data is scheduled.

## 1. Introduction

Preeclampsia (PE) is a clinically severe and potentially life threatening complication of pregnancy. It is characterized by the onset of hypertension (systolic blood pressure ≥ 140 mmHg or diastolic ≥ 90 mmHg) and proteinuria (≥300 mg/24-hour collection) later than 20 weeks of gestation in a previously normotensive woman and occurs in about 2–8% of all pregnancies. It is a progressive disease and delivery cures the mother from symptoms, normally blood pressure normalizes, and proteinuria vanishes within few weeks until 6 weeks [1].

Its exact aetiology and pathophysiology still remain enigmatic [2]. Pathogenesis involves various molecular pathways resulting in endothelial dysfunction which impairs placental function and therefore fetal supply. In addition, maternal kidney endothelial damage leads to a decrease in renal blood flow, glomerular filtration rate (GFR) [3, 4], and proteinuria. The extent of proteinuria is considered as a marker of endothelial dysfunction and therefore of severity of preeclampsia [1, 4–6]. However, microalbuminuria without impairment of renal function has been found several years after pregnancies complicated by PE [7, 8] suggesting persistent endothelial damage at least in some cases.

In this context, epidemiologic studies have demonstrated that preeclampsia predisposes the mother to cardio- and cerebrovascular complications such as hypertension, stroke, or ischemic heart disease [9–13]. On the molecular side various factors also relevant for the development of hypertension in nonpregnant individuals show alterations in PE patients: antiangiogenic soluble fms-like tyrosine kinase-1 (sFlt-1, also known as sVEGFR1) is a naturally occurring, circulating antagonist to vascular endothelial growth factor (VEGF). Increased placental expression and secretion of sFlt-1 appear to play an important role in the pathogenesis of PE as sFlt-1 can be found at increased levels in PE patients [14, 15]. The increase in sFlt-1 serum levels precedes clinical PE by about 5 weeks [16]. Proangiogenic placental growth factor (PlGF) belongs to the VEGF family and is also predominantly expressed in the placenta. PlGF increases in the first and second trimester and decreases in the third and is found at decreased levels in PE patients [17]. The ratio of these two biomarkers (sFlt-1/PlGF) has been recently established as an early predictive marker for the development of preeclampsia in the further course of the pregnancy [18]. Previous studies have discussed the use of the ratio as a possible predictive value to detect the development of preeclampsia, and a cut-off value of 85 had been determined [19]. For a cut-off value of 85, the manufacturer gives a specificity of 95* *% and a sensitivity of 82* *% for their immunoassay.

The purpose of this prospective study was to investigate the predictive value of single prepartum findings indicating severity of preeclampsia for occurrence and extent of impaired postpartum kidney function. Furthermore we assessed the prognostic value of prepartum serum biomarkers sFlt-1 and PlGF for the development of persistent postpartum renal damage in PE patients.

## 2. Patients and Methods

In this prospective, single center study 68 patients with singleton pregnancy between 2012 and 2014 presenting at our department later than 20 weeks of gestational age with elevated blood pressure (systolic blood pressure ≥ 140 mmHg or diastolic ≥ 90 mmHg) and confirmed proteinuria (>300 mg/24-hour collection) were screened.

44 women (Caucasian patients) with preeclampsia meeting inclusion criteria (diagnosis of PE, sufficient knowledge of German language, and having signed informed consent) were included in this study and compared to 39 healthy Caucasian controls (similar gestational week and patient's age). Exclusion criteria were preexisting renal or rheumatologic diseases or diabetes stated in the case history. Also all individuals with a history of preeclampsia in a previous pregnancy were excluded. Also all patients with relevant proteinuria documented in the maternity card before 20 weeks of gestation were excluded. In order to characterize maternal pre- and postpartum kidney function we measured various maternal (blood pressure, proteinuria, creatinine, carbamide, uric acid, serum protein, cystatin c, calcium, and GFR-CDK-EPI) and fetal (birth weight) parameters. Data was collected in both groups not later than 24 hours before delivery. Mothers were scheduled for follow-up examinations at 6 and 12 months including blood pressure measurements and blood and urine analyses (24-hour collection and proteinuria threshold before delivery <300 mg/24-hour collection and after delivery <120 mg/L).

In PE patients maternal venous blood samples for sFlt-1 and PlGF measurements were collected immediately prior to delivery; serum was separated and stored at −70°C until analysis. sFlt-1 and PlGF analysis were performed as an enzyme linked immunosorbent assay (ELISA) as recommended by the manufacturer (Roche Diagnostics, Mannheim, Germany).

This study was approved by the ethical board of Heinrich-Heine-University Duesseldorf and informed consent was obtained from all subjects prior to enrolment.

### 2.1. Statistical Analysis

Data analysis was carried out using R (version 3.1.3) [20]. Boxplots have been created using R package ggplot2 [21]. *p* values below 0.05 are considered statistically significant. The comparison between the PE patients and the controls was entered by the two-sample *t*-test. The correlations are done by Pearson's correlation coefficient. The evaluation of the 95% confidence interval of the correlations was done by Fisher's transformation.

Calculation of regression model for relationship between sFlt-1 and GFR (Figure 4(a)) was done in a two-step process. First, a nonparametric loess regression (using loess from package stats) was calculated allowing nonsupervised calculation of a nonlinear model. Confidence intervals were calculated from standard errors (obtained using predict (…, se = TRUE)) and quantiles of *t*-distribution (as shown in Figure 4(a)). In a second step, a nonlinear least squares regression (NLS) (using nls from package stats) was calculated. An exponential relationship (*y* = *a*exp⁡((−*x*)/*b*)) was assumed after visual fitting of model functions from which also starting parameter estimates (*a* = 8 × 104, *b* = 34) were derived. A numerical simplified version of the parameter estimates is provided. Predictions using simplified parameters differ from predictions using exact parameter estimates less than 1% for GFR > 25 mL/min/1.73 m^2^. The validity of the NLS derived model can be verified using the confidence intervals from loess regression.

## 3. Results

44 patients diagnosed with preeclampsia were compared with 39 gestational weeks and patient's age-matched controls. Compared to controls preeclampsia patients had a significantly increased proteinuria, higher serum creatinine, urea, uric acid, cystatin c, and elevated systolic and diastolic blood pressure. They also had significantly lower glomerular filtration rates (GFR-CDK-EPI), serum protein levels, and fetal birth weights (Table 1) at delivery.

### 3.1. A Proportion of PE Patients Display Persistent Proteinuria and Reduced Glomerular Filtration Rate 6 and 12 Months Postpartum

**Figure 1:**
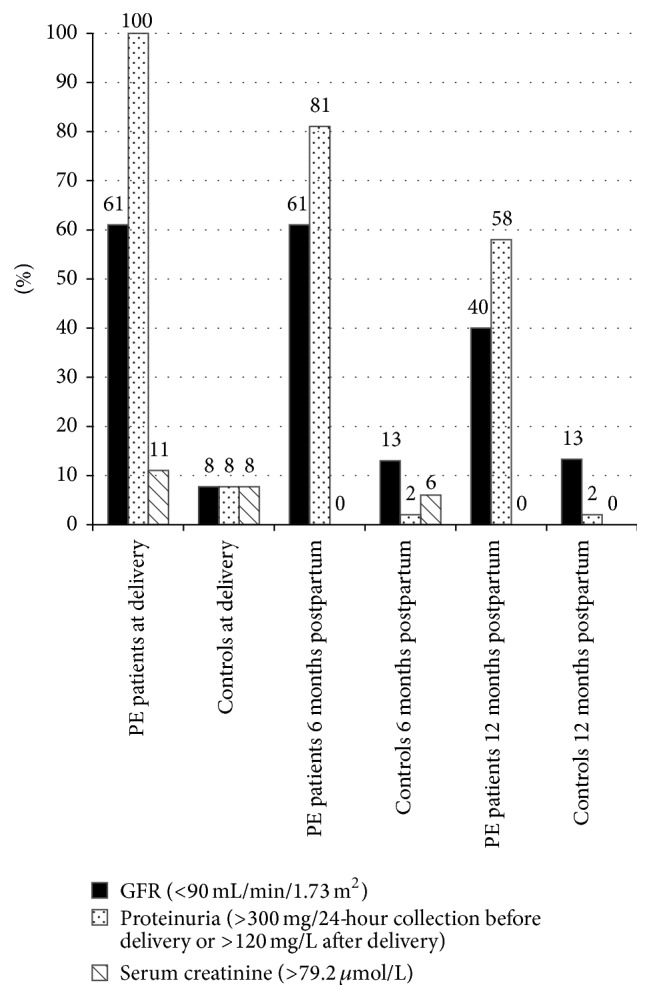
Distribution of impaired renal function parameters (GFR, proteinuria, and serum creatinine) in PE patients and controls at delivery, 6 months postpartum, and 12 months postpartum.

As indicators for impaired kidney function we investigated proteinuria and serum creatinine levels and glomerular filtration rate (GFR-CDK-EPI) (Figure 1). By definition all PE patients had increased proteinuria at delivery (>300 mg/24-hour collection), whereas it was diagnosed in only 3/39 (7.7%) of controls (3/39) (*p* < 0.0001). Reduced GFR < 90 mL/min/1.73 m^2^ at delivery was found in 27/44 (61%) of PE patients and in 3/39 (7.7%) of controls (*p* < 0.0001). Serum creatinine was increased >79.2 *μ*mol/L in 5/44 (11%) of patients and in 3/39 (7.7%) of controls (*p* = 0.72).

A persisting proteinuria (>120 mg/L) was found in 29/36 (81%) of PE patients after 6 months (versus 1/39 (2%) of controls, *p* = 0.02) and 14/24 (58%) after 12 months (versus 2/30 (1%) of controls, *p* < 0.0001). The GFR reduction was found in 22/36 (61%) of PE patients after 6 (versus 5/39 (13%) of controls, *p* < 0.0001) and in 10/25 (40%) of PE patients after 12 months (versus 4/30 (13.3%) of controls, *p* = 0.21), respectively.

### 3.2. Proteinuria and GFR Show a Positive Pre- and Postpartum Correlation

Next we identified predictors of persistently impaired kidney function after 6 and 12 months. Prepartum proteinuria was positively correlated with proteinuria at 6 (*r* = 0.74; 95% CI +0.54 to +0.87) and 12 months postpartum (*r* = 0.55; 95% CI +0.09 to +0.81).

**Figure 2:**
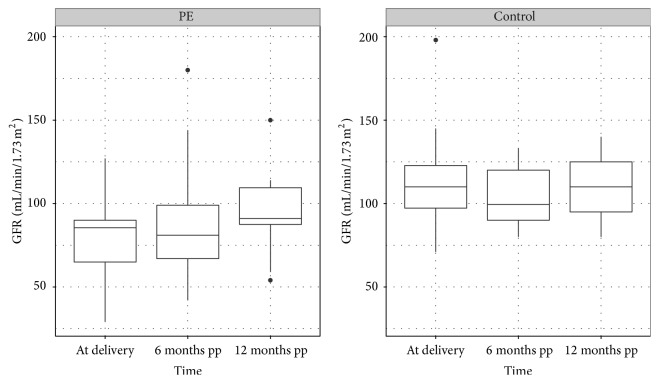
Distribution of GFR in PE patients and controls at delivery, 6 months postpartum, and 12 months postpartum (pp). PE patients: mean GFR 79.3 mL/min/1.73 m^2^ (±23.2 SD) immediately at delivery, 85.4 mL/min/1.73 m^2^ (±28.4 SD) 6 months postpartum, and 95.3 mL/min/1.73 m^2^ (±23.2 SD) 12 months postpartum. Controls: mean GFR 113.0 mL/min/1.73 m^2^ (±22.7 SD) at delivery, 104.0 mL/min/1.73 m^2^ (±15.0 SD) 6 months postpartum, and 110.7 mL/min (±19.3 SD) 12 months postpartum (pp).

We found also a positive correlation between pre- and postpartum GFR in patients after 6 (*r* = 0.73; 95% CI +0.51 to +0.86) and 12 months of (*r* = 0.49; 95% CI +0.01 to +0.78) follow-up (Figure 2). However, prepartum GFR and proteinuria were not correlated (*r* = −0.11; 95% CI −0.39 to +0.20). Also, prepartum GFR was not correlated with postpartum proteinuria after 6 (*r* = −0.22; 95% CI −0.60 to +0.34) or 12 months (*r* = −0.17; 95% CI −0.61 to +0.34), respectively.

The amount of proteinuria at delivery ranged between 300 and 12133 mg/24-hour collection. Proteinuria exceeding 2000 mg/24-hour collection was restricted to PE patients with a GFR < 104 mL/min/1.73 m^2^ and was not found in control individuals.

### 3.3. Negative Relationship between sFlt-1 and GFR

**Figure 3:**
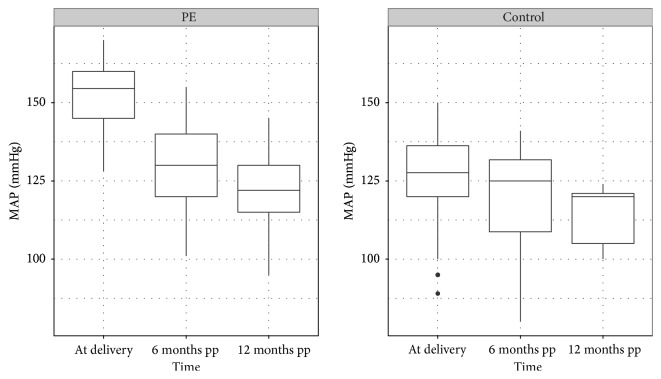
Distribution of mean arterial pressure values (MAP) in controls and PE patients. PE patients at delivery 109 mmHg (±9.6 SD), 6 months postpartum (pp) 100 mmHg (±10.6 SD), and 12 months postpartum (pp) 91 mmHg (±13.6 SD). Controls: at delivery 92 mmHg (±12.6 SD), 6 months postpartum (pp) 93 mmHg (±13.0 SD), and 12 postpartum (pp) 95 mmHg (±7.6 SD).

Prepartum clinical parameters were analysed in order to prove their ability to predict impaired postpartum kidney function. Mean arterial pressure (MAP = diastolic blood pressure + 1/3 *∗* (systolic blood pressure − diastolic blood pressure)) decreased from 109 mmHg (±9.6 SD) prepartum to 100 mmHg (±10.6 SD) at 6 months postpartum (*r* = 0.31; 95% CI −0.04 to +0.59) and to 91 mmHg (±13.6 SD) at 12 months postpartum (*r* = 0.25; 95% CI −0.26 to +0.65) in PE patients. In the controls MAP remained unchanged (Figure 3).

**Figure 4:**
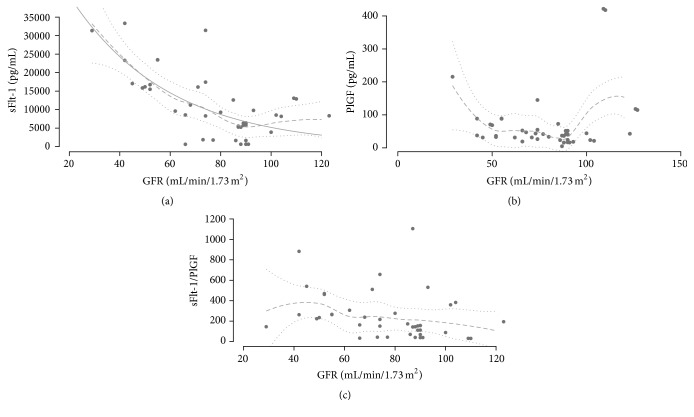
(a) Distribution of sFlt-1 in relation to GFR in PE patients. Predicted values from loess regression are shown together with 95% confidence intervals (discontinuous lines). Predicted values from NLS regression (*y* = *a*exp⁡(−*x*/*b*)) are shown in solid line. Estimated parameter values were *a* = 68169.27  *b* = 38.84402. (b) Distribution of PlGF in relation to GFR in PE patients. Predicted values from loess regression are shown together with 95% confidence intervals (discontinuous lines). (c) Distribution of sFlt-1/PlGF-ratio in relation to GFR in PE patients. Predicted values from loess regression are shown together with 95% confidence intervals (discontinuous lines).

Then we investigated if sFlt-1 or PlGF alone or in combination with each other or other clinical parameters are good predictors of persistent postpartum renal impairment in PE patients. The comparison between GFR and sFlt-1 revealed a significant inverse relationship (*r* = −0.63; 95% CI −0.78 to −0.40). Figure 4(a) shows a relationship between sFlt-1 and GFR values. From nonlinear least squares regression using an exponential model (*y* = *a*exp⁡(−*x*/*b*)) we derived the model

(1) $$\begin{array}{c} y=68170e^{\left(-x/39\right)} \end{array}$$

for relationship between *y* = sFlt-1 (pg/mL) and *x* = GFR (mL/min/1.73 m^2^) using simplified numeric values. Predicted values for sFlt-1 from this model are shown in Figure 4(a) (solid line).

The analysis between PlGF and GFR demonstrated no statistic relevant relationship (Figure 4(b), *r* = 0.16; 95% CI −0.14 to +0.44) and also the analysis between the ratio sFlt-1/PlGF and GFR (Figure 4(c)).

The biomarkers sFlt-1 and PlGF and proteinuria immediately prior to delivery were only weakly correlated (*r* = −0.05; 95% CI −0.34 to +0.25).

**Figure 5:**
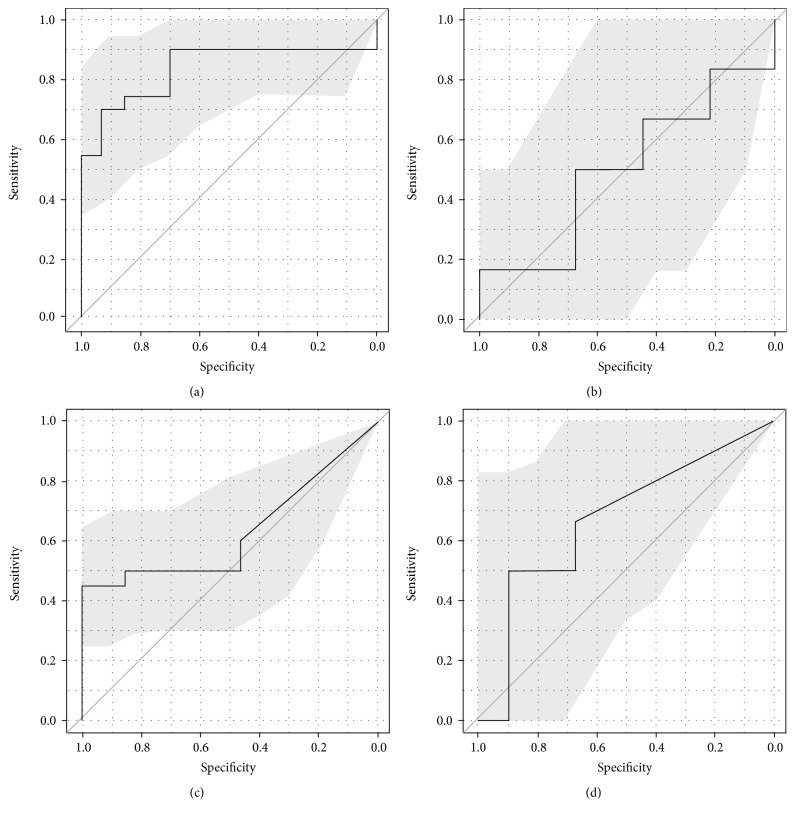
(a) ROC curve for sFlt-1 as a predictive marker for impaired renal function (defined as GFR <90 mL/min/1.73 m^2^) 6 months after delivery in PE patients. The grey shaded area shows the 95% confidence interval. AUC = 0.83. (b) ROC curve for sFlt-1 as a predictive marker for impaired renal function (defined as GFR <90 mL/min/1.73 m^2^) 12 months after delivery in PE patients. The grey shaded area shows the 95% confidence interval. AUC = 0.5. (c) ROC curve for sFlt-1 and MAP >110 mmHg as a predictive marker for impaired renal function (defined as GFR <90 mL/min/1.73 m^2^) 6 months after delivery in PE patients. The grey shaded area shows the 95% confidence interval. AUC = 0.63. (d) ROC curve for sFlt-1 and MAP >110 mmHg as a predictive marker for impaired renal function (defined as GFR <90 mL/min/1.73 m^2^) 12 months after delivery in PE patients. The grey shaded area shows the 95% confidence interval. AUC = 0.67.

In our sample an inverse relationship between sFlt-1 and GFR is present; therefore we analysed all patients with reduced GFR, defined as a GFR below 90 mL/min/1.73 m^2^. sFlt-1 alone reveals a sensitivity of 90% and a specificity of 31% at a value below 5000 pg/mL as a predictive marker for reduced GFR after 6 months postpartum. sFlt-1 values >5000 pg/mL reach meaningful specificities with loss of sensitivity as predictive marker for impaired renal function (Figures 5(a) and 5(b)). Figures 5(c) and 5(d) show also the specificities and sensitivities of sFlt-1 combined with MAP > 110 mmHg as predictive markers for impaired renal function (GFR < 90 mL/min/1.73 m^2^). PlGF did not show any correlation with the investigated parameters.

## 4. Discussion

Improvements in the management of PE patients have lowered fetal and maternal morbidity and mortality caused by PE. Second trimester sFlt-1/PlGF-ratio has the potential to predict PE in asymptomatic patients and therefore to allow early treatment. Early medical treatment and fetal monitoring help to prolong pregnancies in order to avoid increased fetal mortality and morbidity related to preterm delivery and therefore fetal immaturity. In some cases this takes into account that under prolongation of pregnancy PE progresses and therefore maternal morbidity increases. As PE is progressive and delivery cures symptoms for the mother it is crucial to investigate if severity of PE persistently affects mothers' health.

Physiologically, during pregnancy the renal plasma flux and GFR increase until the end of first trimester by 50% and normalize 3 months following delivery [22]. In our controls we also find a moderate increase in GFR during normal pregnancy. In PE placental factors lead to microangiopathy, arterial constriction, microangiopathy, and finally reduced GFR. As expected a significant proportion (61%) of our PE patients had lower GFR levels at delivery, which in 40% persisted up to 12 months after delivery.

One consequence of endothelial dysfunction in PE is the progressive proteinuria, which itself is also an indicator for impaired kidney function beyond pregnancy. In a meta-analysis McDonald et al. combined data from seven cohorts and confirmed the association between PE and persisting albuminuria [23]. At an average of 7.1 years of follow-up, 31% of women who had preeclampsia developed microalbuminuria in contrast to only 7% with uncomplicated pregnancies [23]. In our study also persistent proteinuria was found in 58% of PE patients at least 12 months after delivery but only in 1% of controls. Two other studies showed an increased risk of renal disease after PE [24, 25]. 7.7% of the controls had a relevant proteinuria prepartum but without increase of blood pressure. This proportion is conforming to the present data [24, 25]. There is also evidence that PE is a risk factor for later development of hypertension and cardiovascular diseases [26, 27]. In contrast, persisting proteinuria is not necessarily associated with increased blood pressure or persisting microalbuminuria [28]. Interestingly, in our study sFlt-1 levels correlate with impaired renal function parameters but do not correlate with the extent of proteinuria before delivery of a PE patient compared with healthy controls similar in gestational age or patient's age.

Critically high blood pressure in PE despite medical therapy can be a reason for preterm delivery. This might explain the correlation between increased prepartum blood pressure and lower birth weight and gestational age. High blood pressure during PE pregnancy can also persist [29].

sFlt-1 levels are directly correlated with severity of PE and inversely correlated with time to onset of proteinuria and hypertension in preeclampsia patients [30]. High sFlt-1 levels persist after delivery in preeclampsia patients [31]. We aimed to investigate if sFlt-1 can serve as a predictive marker for persistent endothelial dysfunction and impairment of renal function. There are no sFlt-1 or PlGF measurements in controls prior to delivery, because both parameters are evaluated in preeclampsia patients [19]. We demonstrated a relationship between increased sFlt-1 levels and decreased GFR in PE patients. sFlt-1 is a sensitive prognostic marker for reduced GFR 6 months postpartum below a value of 10000 pg/mL. sFlt-1 values above 10000 pg/mL are attended by a maximum specificity, but lower sensitivity (6 months postpartum). sFlt-1 level until 5000 pg/mL and MAP > 110 mmHg have the most meaningful sensitivities and specificities as predictive markers for impaired renal function (GFR < 90 mL/min/1.73 m^2^) 6 and 12 months postpartum. Overall in our patients collective sFlt-1 is a trend-setting marker in PE, but it is not sufficient enough to predict persistent endothelial dysfunction and impairment of renal function.

Neither sFlt-1 and blood pressure nor prepartum proteinuria alone can predict the extent of renal impairment after delivery. sFlt-1 is indeed a trend-setting marker in PE. Particularly sFlt-1 values until 10000 pg/mL are sensitive to predicting a renal impairment after preeclampsia. Higher values are specific but without sufficient sensitivity. Further investigations are needed to evaluate the prediction of a persisting renal impairment after preeclampsia with angiogenic growth factor sFlt-1.

Limitations of our study are the relatively low number of patients. In an analysis of 30 PE patients with low (<5 g/day) or high (>5 g/day) amounts of proteinuria, proteinuria itself was not correlated with kidney function parameters 5-6 years after pregnancy complicated by PE [32]. We demonstrate a persistence of a clinical relevant proteinuria up to 1 year after delivery in patients with PE. In this respect our study is limited as long term follow-up data is not available. However, an evaluation of 24-month follow-up data is scheduled.

## Figures and Tables

**Table 1:** Baseline characteristics of preeclampsia patients and controls immediately before delivery (maximum 24 hours before delivery). Comparison entered by two-sample *t*-test.

	PE patients (*n* = 44)^*∗*^	Controls (*n* = 39)^*∗*^	Reference values	*p* value
Delivery age (y)	31.95 [22–43]	31.40 [23–42]		0.64
Gestational age (d)	243 [189–278]	245 [195–271]		0.65
Birth weight (g)	2336 [1160–3480]	2720 [1570–3919]		0.003
Proteinuria	2639.9 [301–12133]	180.9 [60–891.5]	<300 mg/24 h in pregnancy <120 mg/L outside of pregnancy	**<0.0001**
Creatinine (*μ*mol/L)	66.88 [35.2–96.8]	52.8 [17.6–105.6]	<0.9	0.001
Urea (mg/dL)	27.41 [10–54]	33.21 [14–55]	21–43	0.018
Uric acid (mg/dL)	6.01 [4.3–8.8]	5.51 [4.1–7.5]	<5.7	0.023
GFR-CDK-EPI (mL/min/1.73 m^2^)	79.32 [29–127]	113.05 [71–198]	90–140	**<0.0001**
Serum protein (g/dL)	5.27 [3.87–6.72]	6.35 [4.7–8.3]	6.40–8.30	**<0.0001**
Cystatin C (mg/L)	1.31 [0.8–2.33]	0.74 [0.66–0.89]	0.6–1.1	**<0.0001**
Calcium (mmol/L)	2.12 [1.7–3.8]	2.08 [1.85–2.51]	2.20–2.55	0.508
sFlt-1 (pg/mL)	10708 [635–33333]	—	—	—
PlGF (pg/mL)	67.9 [5.02–421.5]	—	—	—
Systolic blood pressure (mmHg)	150.95 [128–170]	125.26 [89–145]	<140	**<0.0001**
Diastolic blood pressure (mmHg)	88.79 [67–110]	76.64 [45–92]	<90	**<0.0001**

^*∗*^All values are presented as mean [range].
